# *In vitro* synergistic effect of amlodipine and imipenem on the expression of the *AdeABC* efflux pump in multidrug-resistant *Acinetobacter baumannii*

**DOI:** 10.1371/journal.pone.0198061

**Published:** 2018-06-01

**Authors:** Chao Hu, Yujun Li, Ziwen Zhao, Shuquan Wei, Zhuxiang Zhao, Huiling Chen, Peilian Wu

**Affiliations:** 1 Department of Respiratory Medicine, Guangzhou First People’s Hospital, Guangzhou Medical University, Guangzhou, China; 2 Department of Clinic Laboratory, Guangzhou First People’s Hospital, Guangzhou Medical University, Guangzhou, China; Indian Institute of Science, INDIA

## Abstract

**Introduction:**

Multidrug-resistant *Acinetobacter baumannii* (*A*. *baumannii*) has become one of the greatest threats worldwide to the therapeutic management of infections. Our previous research confirmed an *in vitro* synergistic effect of amlodipine and imipenem against *A*. *baumannii*, and this study is designed to understand its mechanism.

**Methods:**

Sixty-four non-duplicate *A*. *baumannii* isolates were collected and tested for antimicrobial susceptibility by the disk diffusion method. PCR amplification and sequencing were used to identify the presence of the *adeB*, *adeE*, *adeH*, *adeJ*, *abeM* and *abeS* efflux pump genes. The minimal inhibitory concentrations of imipenem, imipenem+amlodipine and imipenem+carbonyl cyanide m–chlorophenyl-hydrazone against these isolates were also determined by the broth microdilution method before and after siRNA silencing of the expression of the adeABC efflux pump.

**Results:**

In this study, the combination of amlodipine with imipenem showed synergistic antimicrobial activity against sixty-four *A*. *baumannii* isolates when compared with the activity of imipenem alone (p<0.025). In the multidrug-resistant group, AML was more effective than carbonyl cyanide m–chlorophenyl-hydrazone (p<0.001). The efflux pump genes *adeB*, *adeE*, *adeH*, *adeJ*, *abeM* and *abeS* were detected in 100% (4/64), 75% (48/64), 0% (0/64), 100% (64/64), 96.9% (62/64) and 96.9% (62/64) of the sixty-four *A*. *baumannii* isolates, respectively. The expression of the *adeABC* efflux pump genes in the multidrug-resistant group (5.05±19.25) is clearly higher than in the non-multidrug-resistant group (0.17±0.20), (p = 0.01). A gene silencing test verified that the mRNA expression levels of *adeABC* were decreased at 12 h and increased at 24 h, while the reversal of imipenem resistance by amlodipine disappeared at 12 h and reappeared at 24 h.

**Conclusions:**

The combination of amlodipine with imipenem exhibits an *in vitro* synergistic antimicrobial effect on multidrug-resistant *A*. *baumannii*, which may be due to the inhibition of the AdeABC efflux pump.

## Introduction

*Acinetobacter baumannii*, a nosocomial pathogen, causes a wide spectrum of healthcare-associated infections including pneumonia, urinary tract infections, septicemia, and wound and skin infections [1]. Because of its intrinsic resistance and its marked ability to acquire and incorporate genetic elements such as plasmids, transposons and integrons that carry resistance genes, multidrug-resistant (MDR) *A*. *baumannii* isolates (i.e., those with resistance to three or more antimicrobial classes) are emerging and spreading worldwide, causing high mortality [2–7]. As microorganisms become increasingly resistant, physicians are encountering increased difficulty in treating resistant infections with the existing antibiotics, and the pipeline of new antibiotics remains lean [8]. *A*. *baumannii* was susceptible to carbapenems in the 1990s but is now exhibiting resistance soon after their initial clinical use. Data from the CHINET surveillance system demonstrated that the resistance of *A*. *baumannii* to many important antimicrobial agents has greatly increased, especially its resistance to imipenem (IPM) and meropenem, which increased from 31% in 2005 to 62.4% in 2014 and from 39% in 2005 to 66.7% in 2014, respectively [9]. Our previous surveillance study indicated that the susceptibility of clinical isolates of *A*. *baumannii* to IPM in our hospital was 25% [10], which was similar to the national survey data mentioned above. The major resistance mechanisms of *A*. *baumannii* to carbapenems involve the production of inactivating enzymes, such as Class B, Class D and Class A β-lactamases, and impermeability combined with the expression of efflux pumps [11,12]. The scientific world is urgently seeking new approaches to overcome the problem of resistance.

Non-antibiotics that act as effective enhancers of existing antibiotic activity, which can reverse antibiotic resistance, have been considered a new hope in the war against antibiotic resistance [13]. Amlodipine (AML), one of the classical calcium channel blockers used to treat hypertension, was recently found to exhibit broad-spectrum antibacterial activities and synergistic antimicrobial effects with a number of antibiotics in many studies [10,14,15]. AML was described as “the most promising cardiovascular antimicrobial non-antibiotic” by Mazumdar et al. [14]. Our previous study demonstrated the antimicrobial activities of AML against clinical isolates of *A*. *baumannii* with minimal inhibitory concentrations (MICs) ranging from 40 to 320 μgml^-1^; AML enhanced the *in vitro* antimicrobial activity of IPM against *A*. *baumannii* isolates but failed to inhibit metallo-β-lactamase (MBL) producers [10]. The mechanism of this phenomenon is unclear. Some reports showed that cardiovascular antimicrobial non-antibiotics such as reserpine and verapamil can inhibit efflux pump systems, blocking signal transduction and drug-resistant mutations [16,17]. An active efflux pump, in combination with mutations to reduce membrane permeability, is considered an important resistance mechanism in *A*. *baumannii*; for example, resistance–nodulation-cell division (RND) efflux pumps (AdeABC) have been found to be linked to IPM susceptibility in *A*. *baumannii* [18,19]. Small interfering RNA (siRNA of 21 to 22 bp) has been used as an important research tool to recognize double-stranded RNA, which may lead to the degradation of target homologous mRNAs upon complementary base pairing [20–22].

According to the aforementioned findings, we hypothesize that AML can inhibit the AdeABC efflux pump of *A*. *baumannii* and thereby reverse its resistance to IPM. This study found the *adeB*, *adeE*, *adeH*, *adeJ*, *abeM* and *abeS* efflux pump genes to be present in MDR *A*. *baumannii* isolates. We also compared the *in vitro* synergistic effects of AML in cooperation with IPM against MDR *A*. *baumannii* isolates before and after siRNA silencing of the expression of the AdeABC efflux pump.

## Materials and methods

### Bacterial strains and drugs

A total of sixty-four non-duplicate *A*. *baumannii* isolates were isolated and collected in Guangzhou First People’s Hospital, Guangdong, China, a tertiary care teaching medical center with 1,571 beds in use, from April 2011 to December 2014. The isolates were obtained as part of the routine activity and were analyzed anonymously in a retrospective manner. The Ethics Committee of the Guangzhou First People’s Hospital approved the study. Document number: K-2017-030-01. Fifty-five of the isolates were MDR, and nine were not (S1 Table). The *A*. *baumannii* isolates were identified with the VITEK^®^ 2 (bioMerieux Inc., Durham, North Carolina, USA) automated microbiology system.

Antibiotic disks (OXOID) and AML were purchased from Melone Pharmaceutical Co. Ltd. (Guangzhou, China). IPM (TIENAM, 500 mg) was obtained from Merck & Co., Inc. (Shanghai, China) and carbonyl cyanide m–chlorophenyl-hydrazone (CCCP) from Sigma-Aldrich Co. LLC. IPM was prepared for antimicrobial susceptibility testing by being dissolved in phosphate-buffered saline (PBS) in accordance with CLSI M100-S22 [23], while AML and CCCP were dissolved in dimethyl sulfoxide (DMSO) [10].

### Antimicrobial susceptibility testing

All sixty-four isolates of *A*. *baumannii* were stored at -80°C and then resuscitated on a blood agar plate and subcultured twice before testing. Disk diffusion susceptibility testing was performed for all the isolates and interpreted according to the CLSI standards [23]. The micro-broth dilution method was also used to determine the MICs of IPM, AML and CCCP alone and in combination against the sixty-four isolates. *Escherichia coli* ATCC 25922 and *Pseudomonas aeruginosa* ATCC 27853 were used as control organisms in each test.

### PCR and nucleotide sequencing

DNA was extracted from the sixty-four isolates by the TaKaRa MiniBEST Bacteria Genomic DNA Extraction Kit Ver.3.0 (TaKaRa Bio Inc., Japan). The presence of efflux system genes (*adeB*, *adeE*, *adeH*, *adeJ*, *abeM* and *abeS*) was detected by PCR with specific primers [24–26] designed and produced by Shanghai Sangon Company (Sangon, Shanghai, China), and the amplicons obtained were sequenced by the same company. The sequence analyses were performed with NCBI BLAST (https://blast.ncbi.nlm.nih.gov/Blast.cgi). All of the primer sequences used in this study are listed in S2 Table.

### SiRNAs and siHybrid transfection

The siRNA sequences designed to interfere with the *adeABC* efflux pump genes in *A*. *baumannii* are described in the previous literature [21,22]. All siRNAs were designed and compounded by RiboBio Co, Ltd. (RiboBio, Guangzhou, China) and are listed in S2 Table. The gene silencing testing was performed in six groups: the three control groups were the sterile growth group, the blank control group and the negative control group, which was named Sc-001, and the three positive siHybrid (si) interfering groups, which were named Si-001, Si-002 and Si-003. The sterile growth group and the negative control group were grown in 3 ml of LB broth medium, while the blank control group was grown in 3 ml of OPTI-MEN-I (growth medium). For each control group, 12 μl of X-treme GENE HP DNA transfection reagent and 120 μl of OPTI-MEN-I were mixed and placed for 5 minutes at room temperature. Then, 5 μl of siRNA and 1863 μl of bacterial suspension (0.5 McFarland) was added to each group, followed by incubation for 12 h and 24 h at 37°C. The MICs of IPM alone and of IPM combined with AML were measured before interference and at 12 h and 24 h after interference. All tests were repeated three times.

### Fluorescence quantitative real-time PCR

The expression of the *adeB* gene in the sixty-four strains was examined by fluorescence quantitative real-time reverse transcriptase PCR (qRT-PCR). The RNA was extracted by using RNAiso Plus (TaKaRa Bio Inc., Japan), and the complementary DNA (cDNA) was obtained by use of the PrimeScript-RT Reagent Kit (Perfect Real Time, TaKaRa, Japan), according to the manufacturer’s instructions. All the real-time PCR assays were performed with Light Cycler 480 Software (Version 1.5) (Roche, Switzerland) and SG Fast qPCR Master Mix (2X) (Bio Basic Inc., Canada). Real-time PCR assays of gene silencing were performed by using Option 2 in the Bio-Rad real-time PCR detection system (Bio-Rad Inc., US) with the SYBR Premix Ex Taq Kit (TaKaRa Bio Inc., Japan). The 16S rRNA gene was used as an internal control to test the expression of the target gene.

### Statistical analysis

SPSS (version 17.0) was used for all of the data analysis. Categorical variables were compared by the Chi-square test with Yates correction or by the Fisher exact test. Continuous variables were analyzed with the t test or rank-sum test. A P-value <0.05 was considered statistically significant.

## Results

### Antimicrobial susceptibility

The antimicrobial susceptibility results of the sixty-four *A*. *baumannii* isolates are shown in S1 Table. Fifty-five MDR *A*. *baumannii* isolates were resistant to ceftazidime, ceftriaxone, aztreonam, levofloxacin, ciprofloxacin and tobramycin, while only a few were sensitive or susceptible to piperacillin/tazobactam (1/55), IPM (5/55) and meropenem (3/55). Table 1 shows the MICs of IPM with and without AML or CCCP. The MICs of IPM alone against all strains ranged from 2 to 64 μg/ml, while in combination with AML or CCCP, the MICs ranged from 1 to 64 μg/ml. In combination with AML or CCCP, the effectiveness of IPM against all strains increased from 25% to 45.31% or 42.19%, respectively (p<0.025, p<0.05). The reductions in the MICs of IPM against the sixty-four *A*. *baumannii* strains due to combination with AML and CCCP are shown in Table 2. In the MDR group, AML was shown to be more effective than CCCP (p<0.001), while in the non-MDR group, there was no significant difference (p = 1.000).

**Table 1:** The MICs of IPM with and without AML (20 μgml^-1^) or CCCP (20 μgml^-1^) in sixty-four strains of *A*. *baumannii*.

drugs	MIC ranges	MIC_50_	MIC_90_	S (%)	Non-S (%)	X^2^ value	P-value
IPM	2–64	8	32	16 (25)	48 (75)	-	-
IPM+AML	1–64	8	32	29 (45.3)	35 (54.7)	5.792^※^	<0.025^※^
IPM+CCCP	1–64	8	32	27 (42.2)	37 (57.8)	4.237^#^	<0.05^#^

S, susceptible; Non-S, non-susceptible

※ (IPM + AML) vs IPM; # (IPM + CCCP) vs IPM.

**Table 2:** Comparison of the reduction in the MICs of IPM in combination with AML and CCCP against sixty-four strains of *A*. *baumannii*.

Groups	AML	CCCP		Statistical analysis	
		(+)	(-)	X^2^ value	P-value
Multidrug-resistant group	(+)	25	7	19.8	<0.001
	(-)	4	19		
Non-multidrug-resistant group	(+)	1	3	0.14	1.000
	(-)	3	2		

(+), reduction in IPM MIC observed; (-), no reduction in IPM MIC observed.

### Detection of efflux genes

**Fig 1:**
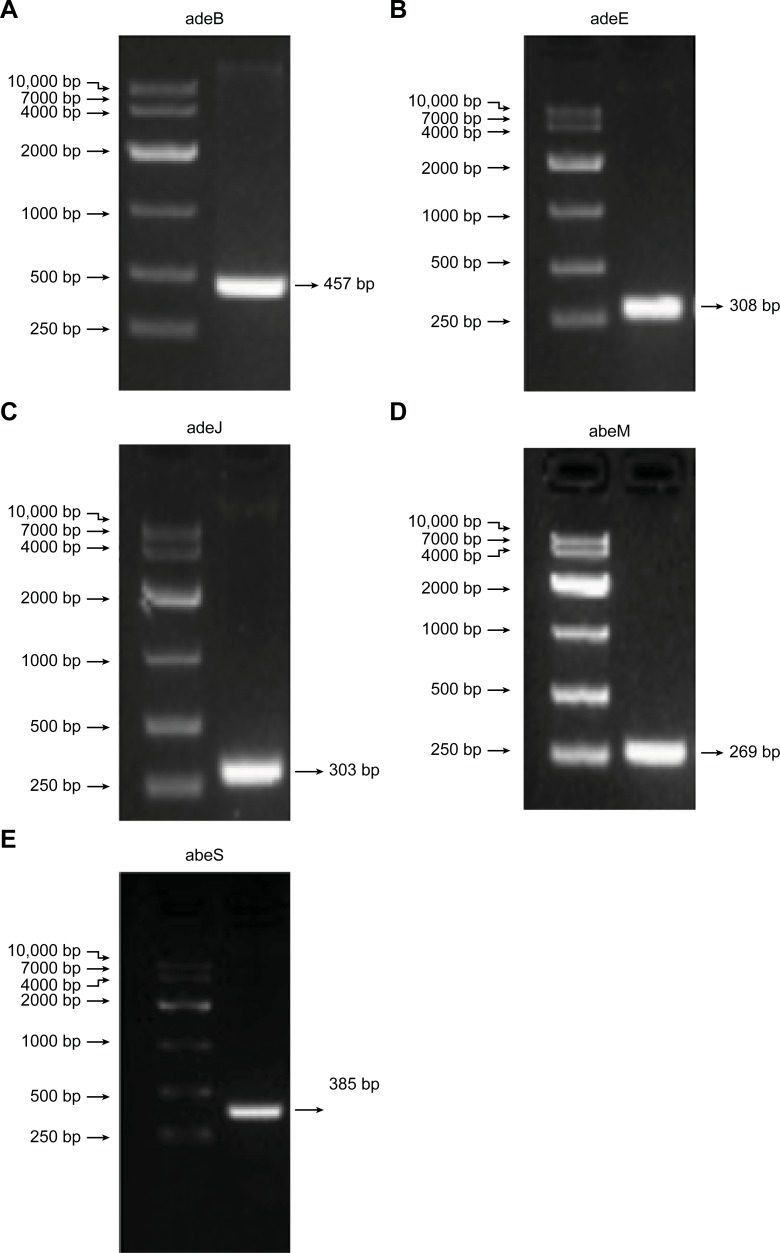
Polymerase chain reaction amplification of the efflux pump gene (adeB, adeE, adeJ, abeM and abeS) of the *A*. *baumannii* isolates. Agarose gel electrophoresis of the product obtained by PCR. (a), adeB gene; (b), adeE gene; (c), adeJ gene; (d), adeM gene; (e), adeS gene; (Left) 10000-bp DNA marker.

Among the fifty-five MDR *A*. *baumannii* strains, the *adeB*, *adeE*, *adeH*, *adeJ*, *abeM* and *abeS* genes were detected in 100% (55/55), 83.6% (46/55), 0% (0/55), 100% (55/55), 96.4% (53/55) and 96.4% (53/55), whereas they were detected in 100% (9/9), 22.2% (2/9), 0% (0/9), 100% (9/9), 100% (9/9) and 100% (9/9) of the nine non-MDR isolates, respectively. Fig 1 shows the electrophoretogram of the PCR amplification of the efflux pump genes.

### Assessment of *adeABC* expression

The differences in *adeABC* expression between the MDR group and the non-MDR group are given in Table 3. As indicated, the expression of *adeABC* in the MDR group (5.05±19.25) was clearly higher than in the non-MDR group (0.17±0.20) (p = 0.011).

**Table 3:** Expression of the AdeABC efflux pump gene in the two groups of *A*. *baumannii*.

Groups	Multidrug-resistant group	Non-multidrug-resistant group	P-value
adeABC	5.05±19.25	0.17±0.20	0.01

**Fig 2:**
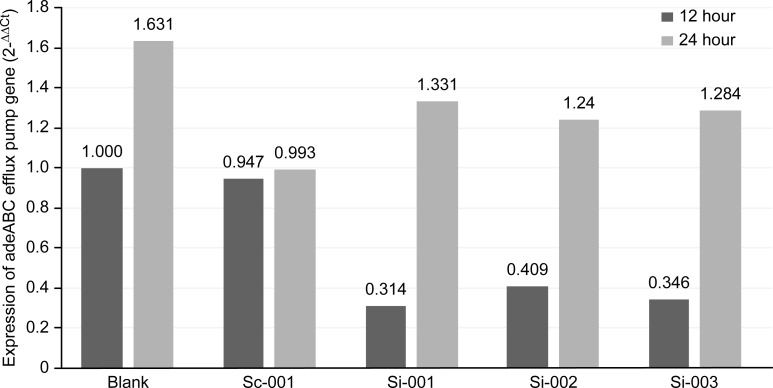
Expression of adeABC gene determined by qRT-PCR of 12 and 24 hours after gene silencing. Blank, blank control group; Sc-001, negative control group; si-001, si-002 and si-003 represent siHybrids interfering group1, siHybrids interfering group2 and siHybrids interfering group3, respectively; Comparison of adeABC gene expressions in three interfering groups between 12 hours and 24 hours, the expression of adeABC efflux pump gene was silenced by siRNA at 12hour but bounced back after 24hour (p = 0.028).

**Fig 3:**
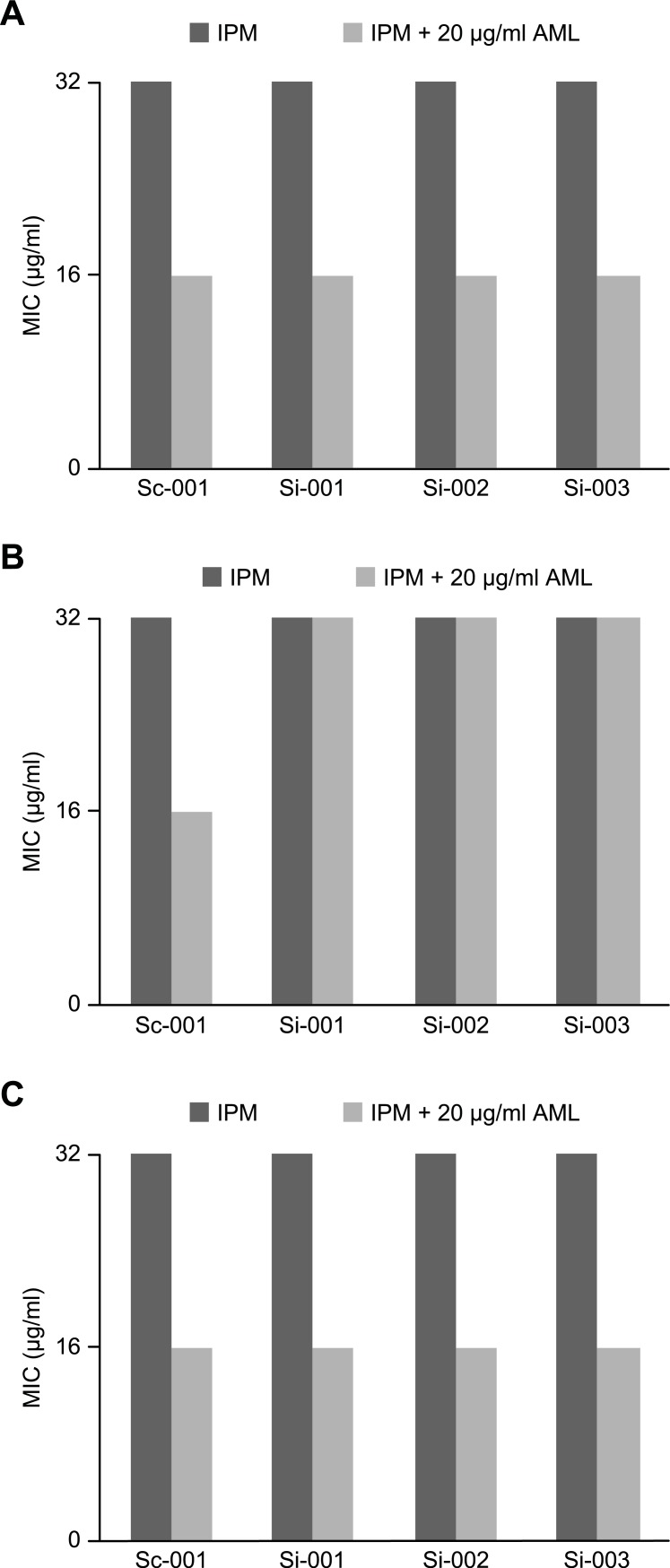
The IPM MICs which against *A*.*baumannii* with and without 20μg ml-1 amlodipine changes during gene silencing process (0, 12 and 24 hours). Sc-001, negative control group; si-001, si-002 and si-003 represent siHybrids interfering group1, siHybrids interfering group2 and siHybrids interfering group3, respectively. There was a reduction of IPM MIC when tested in combination with AML before gene silencing (a). When adeABC efflux pump genes were silenced at 12h, the MIC of IPM combined with AML was not decreased (b). Instead, 24h after the silence, the MIC of IPM combined with AML decreased again (c).

The expression levels of *adeABC* at 12 h and 24 h after siRNA silencing are shown in Fig 2. The expression of the *adeABC* efflux pump was silenced by siRNA at 12 h but bounced back after 24 h (p = 0.028). The 2^-△△Ct^ values and the mRNA expression of the *adeABC* efflux pump genes at 12–24 h are shown in Fig 2. The differences in the MICs of IPM with and without AML against the MDR *A*. *baumannii* isolates before and after gene silencing are shown in Fig 3. The MIC of IPM was decreased when tested in combination with AML before gene silencing (a). When the adeABC efflux pump genes were silenced at 12 h, the MIC of IPM combined with AML was not decreased (b). However, 24 h after silencing, the MIC of IPM combined with AML decreased again (c).

## Discussion

Most *A*. *baumannii* strains in this study were demonstrated to carry the *adeABC*, *adeDE*, *adeIJK*, *abeM* and *abeS* efflux pump genes, which is similar to the results in a previous report [27] and contrary to some former observations [28,29]. These efflux systems are usually intrinsic to *A*. *baumannii* and are overexpressed to cause multidrug resistance after exposure to antibiotics [28]. We found significant differences in the expression of the *adeABC* gene between the MDR and non-MDR groups, which may suggest that the expression levels of the *adeABC* genes are related to the multidrug resistance seen in the study.

Numerous hypotheses have been proposed for the synergistic antimicrobial effect of IPM with AML, such as modifying cellular permeability, curing plasmids, inhibiting efflux pumps and so on. Many calcium channel blockers such as verapamil and reserpine have also been confirmed to have inhibitory effects on the efflux pumps [16,17].

In our study, we found that AML showed synergistic antimicrobial activity in the combination susceptibility testing, as the MIC of IPM decreased when IPM was tested in combination with AML. When sixty to seventy percent of the *adeABC* efflux pump genes were silenced, the MIC of IPM combined with AML was not decreased. However, 24 h after silencing, the MIC of IPM combined with AML decreased again. The above findings showed a strong connection between the *in vitro* synergistic effect of AML and the expression of the adeABC efflux pump.

The MIC of IPM alone remained unchanged when the AdeABC efflux pump was inactivated during the gene silencing test, which appears to contradict the hypothesis. The resistance mechanisms of *A*. *baumannii* are highly complex: in addition to the efflux pump, other resistance mechanisms include Class D β-lactamases, the modification of penicillin-binding proteins and porins [30]. When the AdeABC efflux pump is deactivated, other resistance mechanisms may be reactivated to replace its impact on the antimicrobial activities of IPM against *A*. *baumannii*. The lack of relevant research is an important limitation of the current study. Further research activities will be focused on this area.

Furthermore, the mechanism of the synergistic effect of AML with IMP is intricate and remains largely. AML also exhibits antimicrobial activity against *A*. *baumannii* [10], and many publications have demonstrated that calcium channel blockers can also contribute to improved antimicrobial effects by mechanisms such as changes in membrane permeability and the elimination of biofilms [14,31], which may also impact the antimicrobial activities of IPM against *A*. *baumannii*. However, given that the synergistic effect of AML with IMP appears when the AdeABC efflux pump remains activated, disappears upon deactivation of the AdeABC efflux pump and reappears when the AdeABC efflux pump is reactivated, at least part of the phenomenon involves an inhibitory effect on the adeABC efflux pump.

## Conclusions

AML enhanced the *in vitro* activity of IPM against *A*. *baumannii* isolates, perhaps partly by inhibiting the *adeABC* efflux pump. However, a further comprehensive investigation is warranted. With the coming of the “post-antibiotics era”, more antibacterial enhancer compounds are expected to be discovered to help solve the problems of resistance.

## Supporting information

S1 Table The antimicrobial susceptibility profiles of sixty-four isolates of *A*. *baumannii*, tested by the disk diffusion method and interpreted by the 2012 clsi guidelines [23]. a) The antimicrobial susceptibility results of 55 multidrug-resistant *A*. *baumannii* isolates. b) The antimicrobial susceptibility results of nine non-multidrug-resistant *A*. *baumannii* isolates.
(DOCX)

S2 Table Sequences of the primers [32–34] and siRNAs [21,22] used in this study. (DOCX)
