# Supplementary material for: In vitro synergistic effect of amlodipine and imipenem on the expression of the AdeABC efflux pump in multidrug-resistant Acinetobacter baumannii
Source: PLoS One. 2018 Jun 1;13(6):e0198061. doi: 10.1371/journal.pone.0198061 (PMC5983470; doi:10.1371/journal.pone.0198061)
Supplement: S1 Table — a) The antimicrobial susceptibility results of 55 multidrug-resistant A. baumannii isolates. b) The antimicrobial susceptibility results of nine non-multidrug-resistant A. baumannii isolates. (DOCX) [file pone.0198061.s001.docx]

**S1 Table. The Antimicrobial Susceptibility Profiles of Sixty-Four Isolates of *A. baumannii*, Tested by the Disk Diffusion Method and Interpreted by the 2012 CLSI Guidelines [23].**

a) The antimicrobial susceptibility results of 55 multidrug-resistant *A. baumannii* isolates.

| Isolate no. | Specimen | Ward | TZP | CAZ | CRO | ATM | MEM | LEV | CIP | IPM | TOB |
| --- | --- | --- | --- | --- | --- | --- | --- | --- | --- | --- | --- |
| 1 | Sputum | ICU | R | R | R | R | R | R | R | R | R |
| 2 | Blood | ICU | R | R | R | R | R | R | R | R | R |
| 3 | Sputum | ICU | R | R | R | R | R | R | R | R | R |
| 4 | Blood | ICU | R | R | R | R | R | R | R | R | R |
| 5 | BALF | RICU | R | R | R | R | R | R | R | R | R |
| 6 | Sputum | RICU | R | R | R | R | R | R | R | R | R |
| 7 | Sputum | Respiratory | R | R | R | R | R | R | R | R | R |
| 8 | Sputum | RICU | R | R | R | R | R | R | R | R | R |
| 9 | Sputum | ICU | R | R | R | R | R | R | R | R | R |
| 10 | Sputum | Respiratory | R | R | R | R | R | R | R | R | R |
| 11 | Sputum | Neurosurgery | R | R | R | R | R | R | R | R | R |
| 12 | Sputum | RICU | R | R | R | R | R | R | R | R | R |
| 13 | Sputum | Respiratory | R | R | R | R | R | R | R | R | R |
| 14 | Sputum | ICU | R | R | R | R | I | R | R | I | R |
| 15 | BALF | RICU | R | R | R | R | R | R | R | R | R |
| 16 | Sputum | ICU | R | R | R | R | R | R | R | R | R |
| 17 | Wound | Gastroenterology | R | R | R | R | R | R | R | R | R |
| 18 | Sputum | Respiratory | R | R | R | R | R | R | R | R | R |
| 19 | Sputum | ICU | R | R | R | R | R | R | R | R | R |
| 20 | Urine | ICU | R | R | R | R | R | R | R | R | R |
| 21 | BALF | RICU | R | R | R | R | R | R | R | R | R |
| 22 | BALF | RICU | R | R | R | R | R | R | R | R | R |
| 23 | Sputum | Respiratory | R | R | R | R | R | R | R | R | R |
| 24 | Sputum | ICU | R | R | R | R | S | R | R | S | R |
| 25 | Sputum | RICU | R | R | R | R | R | R | R | R | R |
| 26 | Sputum | RICU | R | R | R | R | R | R | R | R | R |
| 27 | Sputum | Neurosurgery | R | R | R | R | I | R | R | S | R |
| 28 | Sputum | Respiratory | R | R | R | R | R | R | R | R | R |
| 29 | Sputum | RICU | R | R | R | R | R | R | R | R | R |
| 30 | Sputum | RICU | R | R | R | R | R | R | R | R | R |
| 31 | Sputum | Respiratory | R | R | R | R | R | R | R | R | R |
| 32 | Sputum | Neurosurgery | R | R | R | R | S | R | R | S | R |
| 33 | Sputum | ICU | R | R | R | R | R | R | R | R | R |
| 34 | BALF | RICU | R | R | R | R | R | R | R | R | R |
| 35 | CSF | ICU | R | R | R | R | R | R | R | R | R |
| 36 | Sputum | Respiratory | R | R | R | I | R | R | R | R | R |
| 37 | Sputum | ICU | R | R | R | R | R | R | R | R | R |
| 38 | Wound | Burn | R | R | R | R | R | R | R | R | R |
| 39 | Sputum | Gastroenterology | R | R | R | R | R | R | R | R | R |
| 40 | Blood | Urinary Surgery | R | R | R | R | R | R | R | R | R |
| 41 | Sputum | Nephrology | R | R | R | R | I | R | R | S | R |
| 42 | BALF | RICU | S | R | R | R | S | I | R | S | R |
| 43 | Sputum | RICU | R | R | R | R | R | I | R | R | R |
| 44 | Sputum | RICU | R | R | R | R | R | I | R | R | R |
| 45 | Sputum | ICU | R | R | R | R | R | R | R | R | R |
| 46 | Sputum | ICU | R | R | R | R | R | R | R | R | R |
| 47 | BALF | RICU | R | R | R | R | R | R | R | R | R |
| 48 | Sputum | RICU | R | R | R | R | R | R | R | R | R |
| 49 | Sputum | ICU | R | R | R | R | R | I | R | R | R |
| 50 | Sputum | Respiratory | R | R | R | R | R | R | R | R | R |
| 51 | Sputum | Respiratory | R | R | R | R | R | I | R | R | R |
| 52 | Sputum | Respiratory | R | R | R | R | R | I | R | R | R |
| 53 | Sputum | ICU | R | R | R | R | R | I | R | R | R |
| 54 | Sputum | ICU | R | R | R | R | R | R | R | R | R |
| 55 | Sputum | Oncology | R | R | R | R | R | R | R | R | R |

b) The antimicrobial susceptibility results of nine non-multidrug-resistant *A. baumannii* isolates.

| Isolate no. | Specimen | Ward | TZP | CAZ | CRO | | ATM | MEM | LEV | CIP | IPM | TOB |
| --- | --- | --- | --- | --- | --- | --- | --- | --- | --- | --- | --- | --- |
| 56 | Urine | Geriatrics | S | S | S | I | | S | S | S | S | S |
| 57 | Sputum | Cardiothoracic Surgery | S | S | I | R | | S | S | S | S | S |
| 58 | Sputum | Geriatrics | S | S | I | I | | S | S | S | S | S |
| 59 | Sputum | Geriatrics | S | S | I | I | | S | S | S | S | S |
| 60 | Sputum | RICU | S | S | I | R | | S | S | S | S | S |
| 61 | Urine | Geriatrics | S | S | I | R | | S | S | S | S | S |
| 62 | Urine | Geriatrics | S | S | I | I | | S | S | S | S | S |
| 63 | Wound | Neonatology | S | S | I | I | | S | S | S | S | S |
| 64 | Urine | Geriatrics | S | S | I | I | | S | S | S | S | S |

R, resistance; S, sensitivity; I, intermediate; TZP, piperacillin/tazobactam; CAZ, ceftazidime; CRO, ceftriaxone; ATM, aztreonam; MEM, meropenem; LEV, levofloxacin; CIP, ciprofloxacin; IPM, imipenem; TOB, tobramycin
