# Supplementary material for: In vitro synergistic effect of amlodipine and imipenem on the expression of the AdeABC efflux pump in multidrug-resistant Acinetobacter baumannii
Source: PLoS One. 2018 Jun 1;13(6):e0198061. doi: 10.1371/journal.pone.0198061 (PMC5983470; doi:10.1371/journal.pone.0198061)
Supplement: S2 Table — (DOCX) [file pone.0198061.s002.docx]

**S2 Table. Sequences of the Primers [32-34] and siRNAs [21,22] Used in this Study.**

| Gene/ Name | Primer/ siRNA | Sequence (5′-3′) | Product |
| --- | --- | --- | --- |
| *adeB* | B1 | GCTTTACTGGCTGCTCAAGA | 457 bp |
|  | B2 | GTTGTTCCATTTCACGCATT |  |
| *adeE* | E1 | CTTCACCTGCGTTATGTGC | 308 bp |
|  | E2 | TGATTACCAACCCGCTCTGT |  |
| *adeH* | H1 | GCTCCAACACTCAAATCACC | 293 bp |
|  | H2 | ATCACACGGGCACTACAAAC |  |
| *adeJ* | J1 | GCAACAGCCGAAGCAGTTG | 303 bp |
|  | J2 | TGCCAATACCATAGCGAACAT |  |
| abeM | M1 | GCATTGGTCCTTAGCCCTCT | 269 bp |
|  | M2 | TCGGTGCCTGAGTATCTTGC |  |
| abeS | S1 | ATTGCGATTGCTTGTGAAGT | 385 bp |
|  | S2 | ATGCCTGCGATCATTAAAGC |  |
| 16S rRNA | F | ACGGTCGCAAGACTAAAACTCA | 108 bp |
|  | R | GTATGTCAAGGCCAGGTAAGGT |  |
| *adeABC* | F′ | ATGCGTGAAATGGAACAACTG | 147 bp |
|  | R′ | AGCCAAGACAAGGAAGACAACT | None |
| Si-001 | | GCATCTAAAGAGCCTGTAT | None |
| Si-002 | | GCATGTATGTGCGTGTCAA | None |
| Si-003 | | GCATCTAAAGAGCCTGTAT | None |
| Sc-001 | | Not Provided | None |
